# A philosophical perspective on the prenatal in utero microbiome debate

**DOI:** 10.1186/s40168-020-00979-7

**Published:** 2021-01-12

**Authors:** Jens Walter, Mathias W. Hornef

**Affiliations:** 1 APC Microbiome Ireland, School of Microbiology and Department of Medicine, University College Cork – National University of Ireland, Cork, Ireland; 2 Institute of Medical Microbiology, RWTH University Hospital Aachen, Aachen, Germany

## Abstract

Within the last 6 years, a research field has emerged that focuses on the characterization of microbial communities in the prenatal intrauterine environment of humans and their putative role in human health. However, there is considerable controversy around the existence of such microbial populations. The often contentious debate is primarily focused on technical aspects of the research, such as difficulties to assure aseptic sampling and to differentiate legitimate signals in the data from contamination. Although such discussions are clearly important, we feel that the problems with the prenatal microbiome field go deeper. In this commentary, we apply a philosophical framework to evaluate the foundations, experimental approaches, and interpretations used by scientists on both sides of the debate. We argue that the evidence for a “sterile womb” is based on a scientific approach that aligns well with important principles of the philosophy of science as genuine tests of the hypothesis and multiple angles of explanatory considerations were applied. In contrast, research in support of the “in utero colonization hypothesis” is solely based on descriptive verifications that do not provide explanatory insight, which weakens the evidence for a prenatal intrauterine microbiome. We propose that a reflection on philosophical principles can inform not only the debate on the prenatal intrauterine microbiome but also other disciplines that attempt to study low-biomass microbial communities.

## Background

Ignited by a 2014 research study by Aagaard and co-workers that applied next-generation sequencing to describe a unique microbiome in the placenta of humans [1], an entire research field emerged on microbial communities in the fetal environment (placenta, cord blood, amniotic fluid, fetus, meconium) of humans [2–10]. Speculations about the role of these microbial communities, which were often referred to as microbiomes, in initiating the establishment of the human microbiome via in utero transmission and shaping human health were the topic of many commentaries and review articles [2, 3, 5–7]. The findings were big news as they challenged the paradigm of a sterile womb that had been established in the first half of the twentieth century and were widely accepted (as reviewed by Perez-Munoz et al. [11]). Given the immense implications of direct microbial exposure of the fetus for human development and health, scientific journals, funding agencies, and a sizable fraction of the medical community embraced the “in utero colonization hypothesis”. Francis Collins, Director of the National Institute of Health (NIH), enthusiastically supported the concept early on [12], and priorities of funding bodies were changed to accommodate prenatal microbiome research. In both scientific reviews and media reports, intrauterine microbiomes were presented to have far-reaching implications for human health, such as their role in premature birth and infant development [2, 3, 5–7, 13, 14].

However, not everyone shared the excitement. Concerns were raised immediately in a commentary on the Aagaard et al. paper in 2014, which pointed out, among other limitations, that the detection of DNA does not provide evidence for live microbes [15]. The findings were therefore not sufficient to challenge sterility of the womb, as sterility is defined as the absence of viable life. Over the years, it also became increasingly obvious that contamination [16, 17], or the so called “kitome” [18], represented a major problem when next-generation sequencing and PCR-based approaches were applied to low-biomass samples [19]. Consequently, several subsequent sequencing studies that used strict controls for contamination did not support the presence of microbial DNA in utero [18, 20–25]. We, the authors of this commentary here, argued early that the concept of “in utero colonization” was insufficiently supported by the newly created amplification/sequencing data, in our eyes biologically implausible, and in disagreement with a comprehensive body of experimental evidence [11, 26].

Despite the negative findings, the debate has continued and grown constantly more contentious. A recent publication by Rackaityte et al. in *Nature Medicine* reignited the discussion by the provision of evidence for the presence of bacterial DNA and viable bacteria in the fetal intestine, based on 16S rRNA gene sequencing, qPCR, microscopy, and culture data [27]. These findings have now been challenged [28], and we refer readers to this critique and the accompanying response of the authors of the original paper [29], both of which are published in *Microbiome* in parallel to this commentary. These publications, and the ongoing debate in general, are primarily focused on technical aspects of the research, such as the difficulties to differentiate legitimate signals in the data from contamination (which can occur both during sampling and through carry-over of bacterial DNA present in reagents), and the interpretation of such data. Although these discussions are clearly important, we feel they do not cover the full extent of the problem.

In this commentary, we apply a philosophical framework to evaluate the foundations, experimental approaches, and interpretations that have been used by scientists on both sides of the argument to favor one of the two competing hypotheses. We argue that a philosophical reflection can evaluate the scientific assumptions and evidence and inform the debate on the prenatal intrauterine microbiome as well as other low-biomass microbial communities. For clarification, we focus on research that made claims on the existence of microbiomes (specific microbial communities that are in their majority alive and physiologically active) or the colonization of symbiotic microbial species in fetal habitats (niches, sites) in a healthy state. We do not refer to infections with known pathogens or the fetal exposure to microbial constituents and metabolites, for which we think there is strong evidence [20, 24, 30, 31]. We further emphasize that our aim is not to provide a comprehensive overview on biological and technical aspects of the debate, and refer the reader to reviews that have covered these basics [11, 19, 26] and the two accompanying articles published in *Microbiome* [28, 29].

## A philosophical view on the prenatal intrauterine microbiome debate

Philosophy of science is a branch of philosophy concerned with how science should be conducted to contribute to the acquisition of knowledge and guide our thinking of the world to deepen our understanding. Although there is no complete consensus among philosophers about the methodological rules by which scientific research should be conducted, philosophy can judge empirical research through a critique of scientific assumptions, the formulation of hypotheses, and the standards by which they should be tested [32]. In this commentary, we will focus our discussion on two philosophical frameworks; the first put forward by Karl Popper and the second based on the “Inference to the best explanation” framework.

## The prenatal microbiome debate in the light of Karl Popper’s philosophy

Karl Popper is generally regarded as one of the most influential philosophers of science with an outstanding intellectual contribution on how scientific knowledge should be acquired [33]. His books *Logik der Forschung* (published 1959 in English under the title *The logic of scientific discovery*) [34] and *Conjectures and Refutations: The Growth of Scientific Knowledge* [35] describe his “critical rationalism” and the value of falsification over verification. They are considered quintessential contributions to the advancement of scientific inquiry. The reason to focus on Popper is that he is well respected among scientists, and his positions were and remain influential. In *Conjectures and Refutations*, Popper lists seven criteria by which to determine the quality and status of a scientific theory. Below we discuss the scientific evidence used in the prenatal intrauterine microbiome debate in light of these criteria.

*(1) “It is easy to obtain confirmations, or verifications, for nearly every theory—if we look for confirmations”* [35].Verification, or the use of empirical data and observations, has a long history to make rational scientific justification but is problematic for a variety of reasons [36]. Among others, it is logically impossible to generate strong evidence from observations (e.g., inductive reasoning), and verifiable observations do not guarantee correct understanding. In addition, verifications are seldom value-free. Popper’s work was highly influential for early work on the recognition of confirmation bias [37], which is the tendency to search for, interpret, favor, and recall information in a way that confirms or supports one’s preconceived notions. Considering this first principle of Popper, most of the research on the prenatal intrauterine microbiome does not provide strong evidence as it is based on mere verifications that provide perfect conditions for confirmation bias.

The 16S rRNA gene amplification and sequencing approach used in most studies is so sensitive that it detects dozens if not hundreds of microbial taxa no matter if a sample is added or not (the “kitome”) [16, 17]. The approach requires careful sampling, proper controls, bioinformatic tools, and objectivity during the analysis to identify taxa truly overrepresented in samples as compared to controls [18–25, 27, 28, 38, 39]. Microscopy, qPCR techniques, and culture have also been used to confirm the presence of bacteria [27, 38]. Since strictly aseptic sampling is hard if not impossible in a clinical setting [40], the experimental approach will always provide positive findings in a subset of samples if one is keen to find them. On the other hand, negative findings do not provide adequate evidence for absence of microbiomes either, as they might have escaped detection due to inhibitors, populations might be present but under the detection limit, or because criteria for the removal of taxa as contaminants might be too strict. These conceptual and experimental limitations hamper the current prenatal microbiome debate, highlighting the need to employ additional avenues of inquiry that go beyond mere verifications to reach more objective conclusions.

*(2) “Confirmations should count only if they are the result of risky predictions; that is to say, if, unenlightened by the theory in question, we should have expected an event which was incompatible with the theory—an event which would have refuted the theory”*; and *(3) “Every ‘good’ scientific theory is a prohibition: it forbids certain things to happen. The more a theory forbids, the better it is”* [35]. If one wanted to design experiments to generate more objective evidence and test the two competing hypotheses (“sterile womb” versus “in utero colonization”), one could remove the fetus from the womb in a sterile fashion and see if the offspring (i) remains devoid of microbes when raised in a sterile environment, or (ii) becomes colonized by at least some of the taxa reported to be present in the womb. This would satisfy Popper’s demand for *risky predictions* because if just one viable microbe would be present that is able to colonize the newborn, the finding would be incompatible with the “sterile womb paradigm”. Alternatively, success in the derivation of germ-free offspring would be incompatible with the “in utero colonization” hypothesis. In other words, the “in utero colonization” hypothesis would be *prohibitive* of the derivation of germ-free offspring through cesarean-sections, while the “sterile womb” hypothesis forbids the colonization of the offspring by microbes detected in utero.

Although such experiments cannot systematically be done with humans for ethical reasons, they have repeatedly been performed for more than half a century in a wide variety of mammals [41]. Germ-free animals have been generated from cesarean-section born mice, rats, guinea pigs, rabbits, dogs, cats, pigs, lambs, calves, goats, baboons, chimpanzees, and marmosets (reviewed by Perez-Munoz et al. [11]), and the results of these experiments are incompatible with the “in utero colonization” hypothesis. It is unlikely that the microbes reported to be present in the fetal environment would not be able to colonize the offspring gut as germ-free animals provide excellent growing conditions for microbes, including species that have been putatively detected in utero (e.g., *Escherichia*, *Pseudomonas*, *Streptococcus*, *Staphylococcus*, and *Propionibacterium*). In fact, germ-free rodents can even be colonized by microbes that are extremely difficult to culture [42].

One could envision alternative experimental approaches to make “risky predictions,” such as feeding fetal tissues obtained aseptically to germ-free animals, with subsequent testing of colonization. Such experiments would even allow human fetal tissues to be tested, but to our knowledge, such experiments have not been pursued in the prenatal microbiome field. To our knowledge, research on animal models to study the functional consequences and downstream effects of in utero microbial colonization (in the sense of a risky prediction) has not been reported in the peer-reviewed literature. This is disappointing given that an animal model has been developed to study maternal bacterial exposure on the off-spring’s immune development [30]. However, in this model, only metabolites cross the placenta, not complete and viable microbes, which does not support the “in utero colonization” hypothesis.

*(4) “A theory which is not refutable by any conceivable event is non-scientific. Irrefutability is not a virtue of a theory (as people often think) but a vice”*; and *(5) “Every genuine test of a theory is an attempt to falsify it, or to refute it. Testability is falsifiability; but there are degrees of testability: some theories are more testable, more exposed to refutation, than others; they take, as it were, greater risks”*; and *(6) “Confirming evidence should not count except when it is the result of a genuine test of the theory; and this means that it can be presented as a serious but unsuccessful attempt to falsify the theory”* [35]. Popper’s logic of falsification is arguably his most radical concept and has been criticized for a variety of reasons (see section 9 in reference [33]). From a practical standpoint, most scientists do not actively try to falsify their hypotheses (we, the authors, are just as guilty here as others). However, Popper’s ideas were still instrumental to establish one of the most important foundations of science: Hypotheses must be falsifiable, and one cannot regard a proposition or theory as scientific if it does not admit the possibility of being false. Most importantly from a practical perspective, scientists must be open to reject hypotheses in light of evidence and accept the null hypothesis.

Our interpretation of the prenatal microbiome literature suggests that the latter is not the general attitude. Instead, researchers tend to accept the “in utero colonization” hypothesis from simple confirmations or verifications (see #1 above), even if the majority of their own evidence is rather weak and in fact in favor of the null hypothesis. For example, Rackaityte et al. accepted bacterial cell numbers that barely exceeded the detection limit of a qPCR (and which were lower than the cell numbers in two of the procedural sample-negative control swabs) and failed to establish the microbial origin of structures visualized by scanning electron microscopy (which, in our eyes, do not resemble bacteria) [27]. Several of their analyses were performed in subsets of pre-selected samples with no clear indications of how the samples were chosen. According to the authors themselves, only 30% of fetal intestinal specimens produced a bacterial profile different from the controls [27]. Another research group of outspoken advocates of the colonized womb paradigm [5, 9] concluded that findings in their 2020 publication “at the very least support the notion that exposure to bacterial DNA may occur prior to birth in some healthy pregnancies” [8], although less than 20% of the samples contained detectable bacterial DNA. Why would the main conclusion of both publications be based on findings in less than 30% of the samples even though the signals were sparse and despite the limitation that clinical samples cannot be taken aseptically? Admittedly, both publications acknowledge the possibility of contamination. Still, virtually all interpretations of the findings and their implications were made in the context of a colonized womb. We believe that the scientific approach applied in these studies does not represent a rigorous attempt to test the hypothesis and a genuine consideration that it might be false.

*(7) “Some genuinely testable theories, when found to be false, are still upheld by their admirers—for example, by introducing ad hoc some auxiliary assumption, or by re-interpreting the theory ad hoc in such a way that it escapes refutation. Such a procedure is always possible, but it rescues the theory from refutation only at the price of destroying, or at least lowering, its scientific status”* [35]. It speaks for Karl Popper’s understanding of the behavior of scientists (and perhaps human psychology in general) to have made a prediction that fits the response of advocates of the prenatal microbiome to their hypotheses being falsified. Examples of ad hoc axillary assumptions that have been made by scientists in the public press in support of their arguments are listed here:

(i) Babies “come shooting out” too fast to “pick up the mother’s bacteria during birth” [13].
(ii) “It’s awfully darn-tooting hard to make a germ-free animal” and requires “an extreme procedure that would likely remove any resident prenatal microbes” [43].
(iii) “Chemical sterilizing agents that strip the womb of microbes” are used for rodent models that are sterile [44].
(iv) “If we (humans) do not have microbes in utero, we would be the only species that has been interrogated that doesn’t” [45].

None of these ad hoc assumptions are scientifically valid: it only takes seconds to transfer microbes (with plenty of opportunity during the often lengthy process of a vaginal birth), germ-free vertebrates have been generated over the last century without the use of “chemicals that strip the womb”, and *homo sapiens* is not the only species that has been interrogated that does not contain microbes in utero (as demonstrated by 70 years of research with germ-free animals). False statements like these to the public are damaging, and according to Popper, lower the status of the scientific theory they are supposed to support.

## Inference to the Best Explanation (IBE)

Despite providing a well-accepted conceptual template for scientific conduct, some of Popper’s positions, for example his rejection of inductive reasoning and his demand for falsification, have been criticized for setting the bar too high, and for not being reflective of how the majority of day-to-day science is done. Despite its own set of limitations, the vast majority of scientific work (including research in biology and the life sciences) uses abduction to confirm hypotheses [46]. The governing idea is that scientists often do not directly test hypotheses but combine observations with explanatory and mechanistic considerations to choose the hypothesis which would, if correct, best explain the available data, a process often referred to as “Inference to the Best Explanation” [47]. The factors that make one hypothesis more fitting than another may include the quality, depth, and comprehensiveness of the mechanistic evidence.

**Fig. 1:**
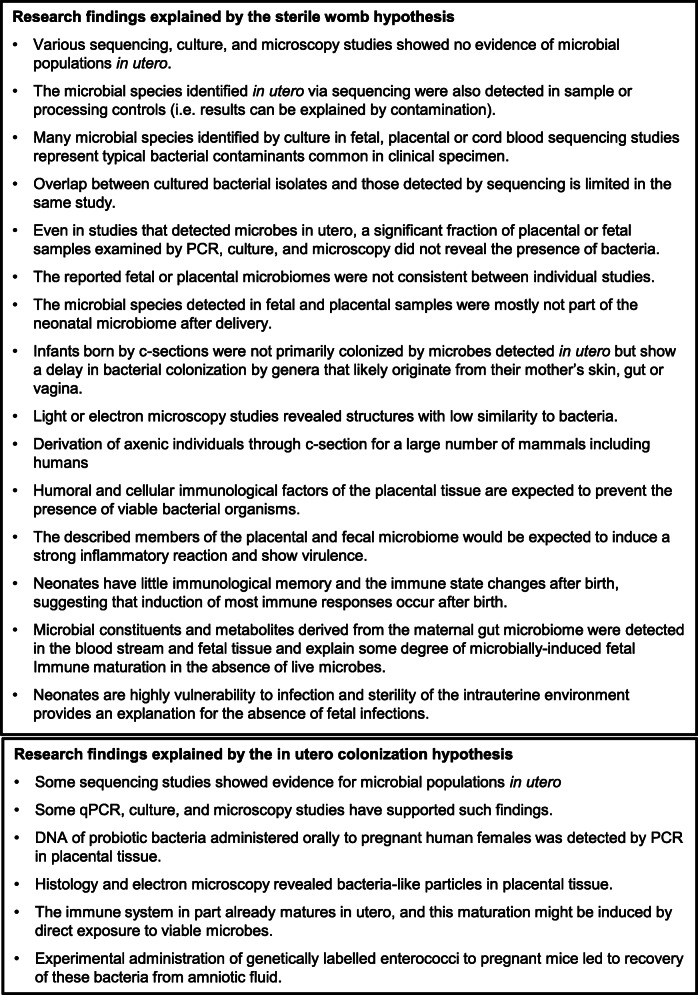
Comparison of research findings explained by the “sterile womb” and “in utero colonization” hypotheses in accordance with “Inference to the Best Explanations” Research findings are presented with the hypothesis they support, with a focus on explanatory and mechanistic considerations. Please see text for references

This philosophical approach does allow a direct comparison of the two competing hypotheses in relation to the supportive data (Figure 1). In addition to the findings from the derivation of axenic mammals and DNA- and culture-based studies that did not confirm microbiomes in utero (see above), findings on the anatomical, immunological, and physiological characteristics of the placenta and fetus as well as the fecal microbiome during the first days of life are in agreement with a sterile womb [11, 26]. For example, in light of the fact that neonates have little immunological memory, a still developing immune system, and an increased vulnerability to infections [48], sterility of the intrauterine environment provides the best explanation for the absence of fetal infections in most pregnancies. Also, the placental tissue has a myriad of antimicrobial mechanisms that should prevent the presence of viable symbiotic microbes [11, 26]. In addition, infants born by c-sections do not primarily become colonized by microbes reported to be present in utero, while they show a delay in bacterial genera that likely originate from their mother’s gut (e.g. *Bifidobacterium* and *Bacteroides*) [49, 50]. For example, gastric aspirates of new-born infants collected immediately after birth do not contain the microbes reported to be present in the amniotic fluid (which would be expected if it were colonized since the fetus swallows amniotic fluid) [51]. Instead, aspirates from vaginal-born infants contain exactly the *Lactobacillus* species that also dominate the microbiota of the vagina (*L. iners* and *L. crispatus*), while most samples from cesarean deliveries cluster with negative controls [51].

Proponents of the colonized womb hypothesis have generally not taken this multi-layered contextual evidence into account and have largely relied on direct sequencing and PCR results. However, there is virtually no agreement between different sequencing studies on which microbes are present in utero, while there is almost complete overlap between the species detected in utero and in contamination controls [11, 18, 20–22, 25]. Many of these species represent typical bacterial contaminants observed in clinical samples obtained under insufficiently hygienic conditions. The microbial populations detected are extremely limited in terms of cell numbers, and microscopy studies only show isolated cells on selected micrographs that do not resemble microbial populations and can be explained by contamination [10, 27, 38]. The explanatory and mechanistic considerations required for IBE are lacking, and critical questions remain unanswered. How do these symbiotic microbial species survive the host’s immune defense mechanisms of the placenta? This is well understood for pathogenic microbes that cross the placenta. How do they avoid strong signs of immune activation and do not induce immunological memory in the neonate [48]? Are these just sparse populations of single persisting microbes or stable and metabolically active communities that qualify as microbiomes? What are the immunological mechanisms that limit the growth of these microbial populations in the fetus, which is especially puzzling given that they expand by at least a million-fold within days after birth? Given the lack of answers to these questions and the comparison of the data in support of the two hypotheses (Figure 1), we and others have come to the conclusion that the “sterile womb hypothesis” is by far the best fit to the available evidence [19, 39, 52].

## How can philosophical considerations inform research on the prenatal intrauterine and other low-biomass microbiomes?

We propose that a philosophical approach can be extremely valuable to inform the contentious debate on the prenatal intrauterine microbiome. As it stands, the sterile womb hypothesis is supported by multiple angles of evidence and explanatory considerations. Having been confirmed by experiments (derivation of axenic animals) that provided genuine tests of the hypothesis by using risky predictions that would have led to its refutation, the sterile womb hypothesis even bears well in light of Karl Popper’s rather strict principles of hypothesis testing. In contrast, research in support of the “in utero colonization hypothesis” has for the most part neglected these principles. By being solely based on descriptive verifications, it largely failed to provide explanatory mechanistic insight. This could, however, change. Although we consider the evidence in support of a sterile womb overwhelmingly strong, the hypothesis remains, of course, falsifiable, and experiments could be designed that go beyond descriptive sequencing studies and provide genuine tests of the two competing hypotheses.

A consideration of philosophical guidelines could also benefit related fields. Dozens of low-biomass microbiomes have been proposed at anatomical sites that were previously extensively studied and considered sterile. Several of them are just as controversial as the intrauterine microbiome in that their sheer existence is questioned (e.g., brain, seminal fluid, breast tissue). Other sites, such as human breast milk, clearly contain microbes, but there are contentious debates about their origin (e.g., entero-mammary transfer, infant’s oral cavity) and their functional and evolutionary roles (autochthonous versus allochthonous) [53]. We provide consideration for research on low-biomass microbiomes in Table 1.

**Table 1:** Suggestions for research on low-biomass microbiomes considering philosophical principles

1. **Rationale for the study.** It is legitimate to challenge dogma but published evidence that led to the dogma in the first place should not be ignored but incorporated and explained. If such published data does not support the hypothesis, and there is no good reason to question the quality without making ad hoc auxiliary assumptions, strongly consider if the project has merit. Microbiology is an old discipline that dates back more than 100 years, and the more traditional methodologies such as culture and microscopy are, in many cases, legitimate approaches to determine the presence of microbial communities. Immunological assays represent complementary tools to functionally test the presence of microorganisms. Only because a technology is new and exciting does not necessarily mean it is superior to the traditional approaches. 2. **Use appropriate methodology and try to extend beyond descriptive studies that are mere verifications.** DNA- or RNA-based next-generation technologies, as powerful as they are to study complex microbial communities, have immense limitations for the study of low-biomass samples due to contamination issues. DNA-based methods are also not suitable to establish sterility as they do not assess viability. Classical approaches, such as culture, and microscopy to detect nonculturable microbes, are better suited to establish the presence of microbes as they are less susceptible to contamination. With the right microbiological expertise, it is not hard to establish the existence of entire microbiomes. Sequencing can be used as a follow-up after the presence of microbes is clearly established to determine which microbes are there. Other techniques, such as microscopy using selective labels for bacteria or specific taxa, should also be employed. For all techniques, aseptic sampling remains a major challenge, and appropriate contamination controls must be employed. Admittedly, initial studies in any field are often exploratory studies that are descriptive in nature and represent verifications. Subsequent studies should, to be truly insightful, go beyond this and try to provide explanatory and mechanistic insight in accordance with IBE. 3. **Study the broader biological context.** It is imperative for future studies to strictly differentiate between viable symbiotic microbiomes/microbes, pathogens, and microbial metabolites/products, as they would differ in their functionality, biological effects on the host, and in the experimental approaches by which they would have to be studied. Extend purely descriptive investigations with experiments that allow a broader interpretation of the findings considering the overall biology, ecology, and evolution of the biological system. For example, if bacterial sequences are detected in utero, are the same bacterial strains detectable in the infant gut? If there are bacteria detected in breast milk, determine if they really have an evolved role in establishing the infant’s gut microbiome, or are they just reflective of the mother’s skin microbiota or the infant’s oral microbiota inoculating the breast milk [54]? Are bacteria in the brain not prevented by the immune response they would elicit? Can the expected immune response be detected? Consider various angles of evidence, especially those that provide explanatory and mechanistic information, in accordance with IBE. 4. **Conduct studies that involve genuine tests of falsifiable hypotheses**. Make efforts to design studies that test hypotheses through “risky” predictions that are genuine tests of conditions that are prohibited by the theory. As a minimum, hypotheses must be falsifiable and refutable, and they should be rejected if proven false without using ad hoc auxiliary assumptions to make the data fit the theory. 5. **Avoid hype**. It is legitimate to challenge dogma, but it requires “extraordinary evidence to back up an extraordinary claim” [52]. Experimental and technical limitations of any study should be acknowledged and unsubstantiated claims about the significance of the data avoided. Be especially careful with unsubstantiated claims on mainstream media as journalists tend to exaggerate the importance of scientific findings and remain critical and balanced with statements on social media.	

## Conclusions

Controversies are an integral part of the scientific process, and studies that challenge scientific dogma are necessary and often beneficial. However, as it relates to the research in the prenatal fetal microbiome, we think much has been going wrong, especially in terms of the uncritical acceptance of premature findings, the emphasis on novelty at the expense of rigor, and how findings and their implications were reported to the public. One could argue that the 6 years since the first publication [1] is not a long period by scientific standards and that the current debate is sign of science correcting itself. However, the debate on the sterility of the womb goes back more than a hundred years [11], and we think it is fair to pose the question how it re-emerged although the ultimate (in Popper’s words, “risky”) experiments to disprove its overall premise have continuously been done for 70 years. Tens of millions of dollars have been spent to investigate microbial populations that likely do not exist, money that could have been used to study more plausible aspects of the prenatal microbiome, such as the role of pathogens, microbial products, and metabolites from the maternal microbiome in fetal and intrauterine sites [20, 24, 30, 31].

The negative impact of this controversy on scientific credibility, and the public’s trust in science, is not negligible. Despite the lack of understanding of the limitations of the reported data and the unresolved experimental, analytical, and conceptual questions, the intrauterine microbiome was communicated to the public as a blockbuster discovery on what should be reliable channels of information, such as the NIH Director’s blog and the mainstream news [12–14]. Some years later, the lay public got informed that not only was none of the early hype justified [55], but that experts even disagree on just the sheer existence of entire microbiomes at a particular location despite significant public-funded research. Such messages could have lasting effects on the public trust in the scientific process, especially since the scientific self-correction process is now much slower than the transfer of information.

We are convinced that much of this could have been avoided with a stronger emphasis on philosophical reflections on how science should be conducted, interpreted, and reported. Even should the reader disagree with us on the scientific specifics of this debate, we at least hope we have provided compelling arguments for a consideration of philosophical principles in the critique of scientific assumptions and to guide future research. As it relates to the prenatal intrauterine microbiome debate, we think that the evidence for the sterile womb is overwhelmingly strong. But as Popper pointed out, “we realize also that we can never be completely certain” [56].

## Data Availability

Not applicable
